# Sheffield Voluntary Hospitals Committee

**Published:** 1922-02

**Authors:** S. R. Lamb

**Affiliations:** Secretary


*Secretary.*

### Sheffield Voluntary Hospitals Committee.

*To the Editor of* The Hospital and Health Review.

Sir,—It may be of interest to you to know that, as the result of recent negotiations with the Ministry of Health, the following Committee of the Sheffield Hospitals' Council has been authorised by the Voluntary Hospitals' Commission to be the Local Voluntary Hospitals' Committee for this area :—

Representing the City Corporation—
- The Lord Mayor (Ald. Chas. Simpson) ;
- Ald. W. F. Wardley, J.P. (Secretary, Cutlers' Union).

Representing the Sheffield University—
- Sir Henry Hadow, C.B.E. (Vice Chancellor).

Representing the General Hospitals—
- Mr. H. H. Bedford, J.P. (Chairman, Royal Infirmary).

Representing the Special Hospitals—
- Mr. T. Walter Hall (Vice Chairman, Sheffield Hospitals' Council).

Representing the Hon. Consultants—
- Dr. Arthur Hall.

Representing the Medical Practitioners—
- Dr. A. Forbes.

Nonimated by the Voluntary Hospitals' Commission—
- Councillor T. Driver ;
- Mr. W. E. Hart (Town Clerk) ;
- Mr. R. W. Fowler (Chairman, Children's Hospital)
- Mr. F. M. Osborn ;
- Councillor Mrs. Wilkinson (District Secretary, Federation of Women Workers.)

This Committee, under the direction of the Sheffield Hospitals' Council, is to carry out the functions suggested by the Voluntary Hospitals' Commission.

Faithfully yours,

S. R. Lamb,

*Secretary.*

### THE COLLEGE OF NURSING.

Nurses throughout the country have desired to make a wedding present to Princess Mary as a personal gift. They will be pleased to learn that, on being approached by the College of Nursing, Her Royal Highness replied that she was much touched by the kind offer and would be delighted to accept the gift. No doubt many nurses will not wish to be limited to any precise sum, but in order to give an opportunity to all to contribute, the Secretary of the College of Nursing will be glad to receive One Shilling from each nurse or nurse in training, and a postcard acknowledgment will be sent from 7, Henrietta Street, Cavendish Square, W.1.

